# Natural Compounds with Beneficial Effects on Skin Collagen Type I and Mechanisms of Their Action

**DOI:** 10.3390/antiox14040389

**Published:** 2025-03-26

**Authors:** Wioleta Żynda, Agnieszka Ruczaj, Anna Galicka

**Affiliations:** Department of Medical Chemistry, Medical University of Bialystok, ul. Mickiewicza 2A, 15-222 Bialystok, Poland; wioleta.zynda@umb.edu.pl (W.Ż.); agnieszka.ruczaj@umb.edu.pl (A.R.)

**Keywords:** collagen type I, skin, fibroblasts, aging, polyphenols

## Abstract

The skin, as the largest external organ, is exposed to many environmental factors, such as sunlight and pollution, as well as some synthetic ingredients in cosmetic products used in excess by most people of all ages throughout their lives. Under the influence of these factors and with age, the amount of the key building protein, collagen type I, decreases, which leads to a deterioration in the appearance and condition of the skin. Currently, when the average life expectancy increases, the esthetic aspect and maintaining healthy skin are particularly important. In the cosmetic and pharmaceutical industries, attempts have long been made to prevent skin aging by the application of products containing natural compounds, mainly due to their high antioxidant activity. This review collects natural compounds, mainly polyphenols, with stimulating and protective effects on collagen type I in human skin fibroblasts, along with a description of the mechanisms of their action. Some of them have been tested on mice and rats, as well as in clinical trials, and in most cases, the results have been very promising. Nevertheless, there is still a need for an intensification of clinical studies in order to determine their appropriate dosage, safety, and effectiveness.

## 1. Introduction

Collagen type I, composed of two α1 and one α2 chains, is the most abundant component of the extracellular matrix (ECM) of the connective tissue [1,2,3]. It occupies over 90% of all (28 types) collagens in the human body, and it is a major structural protein in the skin (80–85% of the dermal ECM), bone (90% of the organic mass), tendons, ligaments, cornea, and many internal organs such as the liver, lungs, and heart [4,5]. Other collagens found in the skin in smaller amounts include collagen type III (8–12%) and type V (5%) as fibrillar collagens and collagens associated with fibers (type XII, XIV, XVI, and VI) [6,7]. Collagen type I, as the key fibrous component, is responsible for maintaining the structural integrity, stability, and tensile strength of the connective tissue [1,7,8]. As the basic building protein of the skin, it is crucial for the skin’s health and contributes to providing its mechanical strength, elasticity, and smooth appearance [9,10]. In addition to its structural role in the assembly and organization of the ECM and giving the tissues mechanical properties, collagen type I has an important function in cell adhesion, the regulation of cell proliferation and migration, as well as cell–matrix interactions and cell signaling [11,12]. Type I collagen can bind inflammatory interleukins (IL-1, IL-6, and IL-8) and create a physiological wound environment that supports the healing process [4,6,13]. Its angiogenic activity is dependent on binding to integrin receptors (α1β1 and α2β1) on the surface of endothelial cells [12,14].

The content of type I collagen declines with age, but there are many endogenous and exogenous factors that can accelerate this process. One of these is photoaging [15,16]. Ultraviolet (UV) radiation, generating reactive oxygen species (ROS), stimulates skin inflammation and activates transcription factors that regulate the degradation of the skin ECM. Type I collagen is then degraded to a greater extent by matrix metalloproteinases (MMPs), which belong to the family of ubiquitous endopeptidases that also degrade other ECM components [17]. In aging skin, in addition to quantitative changes resulting from reduced collagen biosynthesis and increased degradation, structural changes also occur in collagen fibers, as they become fragmented and coarsely distributed [15,16,18]. Abnormal collagen homeostasis occurs not only in naturally aging skin and skin exposed to UV radiation but also when it is chronically exposed to various environmental pollutants, heavy metals (tobacco smoke) [18,19,20], or some of the ingredients of everyday cosmetic products (such as parabens and UV-filters) [21,22,23].

A disruption in the homeostasis of the basic building component of the skin ECM accelerates the appearance of clinical changes in the skin, such as a decrease in skin thickness and wrinkles, age spots, and the loss of skin tone, integrity, and elasticity as the skin loses its tensile strength [9,15,16,19,20]. In addition to the worsening appearance of the skin, the skin functions also become weaker. The above-mentioned symptoms of skin deterioration are the result of not only collagen type I metabolism alterations but also other important building components of the skin ECM, including proteoglycans and glycosaminoglycans (hyaluronic acid—HA), which are primarily responsible for maintaining proper skin hydration, and elastin, which together with collagen ensures skin elasticity [4,9,24].

In recent years, there has been a growing interest in anti-aging collagen supplements containing collagen from marine fish, which shows significant homology to human collagen, but due to its high molecular weight, it does not penetrate the skin [25]. A key strategy for protecting the skin and delaying the aging process is the use of natural compounds that stimulate the biosynthesis of this key protein. The application of preparations containing natural compounds directly to the skin can stimulate collagen biosynthesis, prevent its degradation by inhibiting the activity of MMPs, and bring beneficial effects to the skin. In connection with the above, the search for and implementation of substances with such effects in clinical and cosmetic practice have become the leading topics of many scientific studies. Since oxidative stress is a key factor affecting collagen and responsible for the skin aging process, most therapeutic strategies focus on low-molecular-weight polyphenolic compounds with high biological activity, characterized primarily by strong antioxidant properties [26,27,28,29,30].

This review is a summary of polyphenols and other natural compounds with stimulating and protective effects on type I collagen, along with their mechanisms of action. Their simultaneous beneficial effects on the skin are emphasized, which can undoubtedly support their therapeutic potential, and examples of their effects in clinical studies are given.

## 2. Collagen Biosynthesis and Regulation

Collagen biosynthesis is a complex process involving the transcription of collagen genes, the translation and translocation of the nascent procollagen chain to the rough endoplasmic reticulum (rER), post-translational modifications and triple-helix formation, transportation through the Golgi network, secretion, extracellular processing, and finally, the process of collagen fibrillogenesis [1,5,8,31,32]. This process will be briefly described for type I collagen, which belongs to the major fibrillar collagens. After the transcription of the *COL1A1* and *COL1A2* genes, mRNA is translated into the procollagen chains α1 and α2, respectively, at the rER. Before forming the triple helix, the polypeptide chains undergo important modifications involving many enzymes and molecular chaperones. First, the N-terminal signal peptide is removed by signal peptidase, and three procollagen chains assemble into trimeric collagen monomers, stabilized by an intrachain disulfide bond in the C-propeptide region. In this way, the process of triple-helix formation is initiated toward the N-terminus. Free procollagen chains not included in the triple-helix structure undergo the enzymatic hydroxylation of specific proline and lysine residues catalyzed by prolyl 4-hydroxylase, prolyl 3-hydroxylase, and lysyl hydroxylase [33]. Hydroxyproline residues contribute to the thermal stability of the triple-helix domain by forming intramolecular hydrogen bonds [33]. Hydroxylysine residues participate in the formation of intermolecular covalent cross-links in the process of collagen fiber formation, and some of them also undergo glycosylation [8,31,32]. The enzymes hydroxylysyl galactosyltransferase and galactosyl-hydroxylysyl-glucosyltransferase transfer glucosyl and galactosyl residues to the hydroxyl groups of some hydroxylysines. Intracellular processes involving modifications and the folding of procollagen chains into a triple helix are supported by many enzymes and chaperones, including Grp78 (BiP), Grp94, protein disulfide isomerase (PDI), and cyclophilin (CypB) [34,35].

The right-handed superhelix, which is formed by winding three left-handed polypeptide chains around its own axis, is a characteristic feature of the structure of collagen. The formation of the triple helix proceeds in a zipper-like manner, and the cis–trans isomerization of proline residues is important in this process [1,2,6,31]. This reaction is catalyzed by peptidyl-prolyl cis–trans isomerases including FKBP10 (FKBP65), CypB, and FKBP14 (FKBP22). In addition, heat shock protein 47 (Hsp47 or SERPINH1) as a type I collagen-specific chaperone plays a critical role in the correct folding of procollagen. It also protects the collagen helix structure and prevents fibril formation and aggregation during the export of procollagen from the ER to the Golgi apparatus [34,35,36].

Collagen is a secretory protein that requires the action of many proteins in this process: TANGO1, cTAGE5, Sedlin, and Sec proteins [36], which ensure efficient sorting into coat protein complex II transport vesicles. During the sorting step, the chaperone Hsp47 acts as an anchor molecule between TANGO1 and type I procollagen [34,35,36].

After the secretion of procollagen, N-propeptides and C-propeptides are cleaved by metalloproteinases, namely N-protease ADAMTS and C-protease BMP1, which results in the formation of collagens. Fibrillar collagens such as type I collagen, after the step of the cleavage of propeptides, spontaneously, entropy-driven, associate to form fibrils [8,31,32,33]. They are stabilized by intra- and intermolecular cross-links between lysine and hydroxylysine residue, catalyzed by the lysyl oxidase and transglutaminase family. Covalent cross-links are responsible for the formation of strong collagen fibrils, which combine into fibers and determine the mechanical strength of the skin [37,38].

As collagen biosynthesis is a complex process, it is also subject to complex regulations at different intra- and extracellular stages involving many factors [31,39]. One of the most important factors controlling collagen homeostasis in human skin fibroblasts is transforming growth factor β (TGF-β), which affects collagen biosynthesis and degradation via the suppressor of mothers against decapentaplegic (Smad)-dependent and independent pathways. TGF-β signaling is mediated by its receptors (type I (TβRI) and type II (TβRII), as well as type III, which act as co-receptors) [40,41]. The receptors are transmembrane serine/threonine kinases that, when they bind to TGF, form ligand–receptor complexes. After binding to the TβRII, TGF-β recruits and phosphorylates TβRI through the receptor II kinase. TβRI transmits the signals into the cell, leading to the activation of the transcription factors Smad2 and Smad3 through their phosphorylation. Activated Smad2 and Smad3 form heteromeric complexes with Smad4 and translocate to the nucleus, where they interact with Smad-binding elements in the promoter regions of the target genes [40,41]. Type I collagen genes are upregulated, while genes encoding enzymes (MMPs) responsible for collagen degradation are downregulated. Smad7, in turn, inhibits collagen biosynthesis via a feedback inhibition mechanism, reducing Smad2/3 activation and TGF-β receptor stability. Any factors disrupting the functioning of the TGF-β/Smad pathway are associated with abnormalities in collagen metabolism [40].

Moreover, TGF-β can control the biosynthesis of type I collagen through pathways that do not involve Smad, including the mitogen-activated protein kinase (MAPK) pathways or protein kinase B (Akt) signaling [42,43]. In dermal fibroblasts, TGF-β promotes extracellular signal-regulated kinase (ERK) signaling, leading to increased collagen biosynthesis [43]. A reduction in the Akt-related phosphoinositide 3-kinase (PI3K)/Akt/mammalian target of rapamycin (mTOR) signaling results in the inhibition of collagen biosynthesis and the increased expression of MMPs [44].

Insulin-like growth factor 1 (IGF-1) is the primary growth factor responsible for inducing collagen biosynthesis in dermal fibroblasts [45]. IGF-1 signal transduction is mediated through the IGF-1 receptor (IGF-1R), which is a tyrosine kinase consisting of transmembrane glycoproteins α and β. Upon ligand binding, the receptor undergoes dimerization and activation and phosphorylates several substrates such as insulin receptor substrates (IRS-1 and IRS-2), and Src homologous and collagen-like protein Shc. The phosphorylated residues are recognized by the signaling molecules p85 and Grb2, which stimulate the PI3K and MAPK signaling cascades. These signaling cascades mediate the crucial biological functions of IGF-1 [45,46].

The stimulating effect on collagen biosynthesis is also demonstrated by the fibroblast growth factor, the epidermal growth factor, and various isoforms of the platelet-derived growth factor. Factors that negatively regulate collagen include interferons (IFN-α and IFN-γ), the tumor necrosis factor (TNF), and MMPs [31,39].

## 3. Natural Compounds with Stimulating and Protective Effects on Skin Collagen Type I

Polyphenols are of increasing interest to scientists due to their high biological activity and beneficial effects on human health [47,48]. They are the secondary metabolites of plants that participate in defense against UV radiation and pathogen aggression. They are found mainly in herbs, fruits, vegetables, cereals, propolis, and beverages including juice, tea, coffee, and wine [47,49]. Numerous studies conducted in in vitro and in vivo have shown their strong efficacy in the therapy of numerous diseases such as diseases of the circulatory system, diabetes, neurodegenerative and degenerative diseases, inflammatory conditions, autoimmune diseases, obesity, and cancer [47,48,49,50]. For a very long time, natural products, especially plant products, have been used to improve or eliminate the undesirable signs of aging skin; hence, they are common ingredients in many pharmaceutical and cosmetic products. They exhibit multifaceted effects such as antioxidant, anti-inflammatory, antibacterial, antiviral, anti-wrinkle, moisturizing, skin whitening, UV absorbing, and anticancer [26,27,28,29,30]. This high biological activity of polyphenols is of great importance in protecting the skin from infections and many external factors (UV, smoking, and pollution) that negatively affect the skin barrier function. In addition to stimulating and protecting the type I collagen properties, they prevent other ECM components, e.g., hyaluronic acid, from excessive degradation, thus maintaining proper hydration of the skin [27]. Some polyphenols, e.g., cyanidin-3-O-glucoside, belonging to anthocyanins, protect HDFs against photoaging by activating autophagy [26]. Many compounds from different groups of polyphenols have antimutagenic and anticancer properties [28]. Apigenin showed a protective effect on HDFs against UVB-induced cyclobutane pyrimidine dimer formation, nuclear fragmentation, and increased apoptosis. Moreover, it strongly absorbed UV radiation [26]. Others accelerate DNA repair in UVB-damaged HDFs [28]. Many natural compounds (including ellagic and ferulic acids) have whitening properties, widely used in cosmetic treatments, because skin pigmentation increases with age, under the influence of UV radiation, or can be a result of endocrine disorders [29]. These examples demonstrate the additional valuable activities of natural compounds that stimulate the biosynthesis of type I collagen, which can enhance the benefits for the skin.

**Figure 1:**
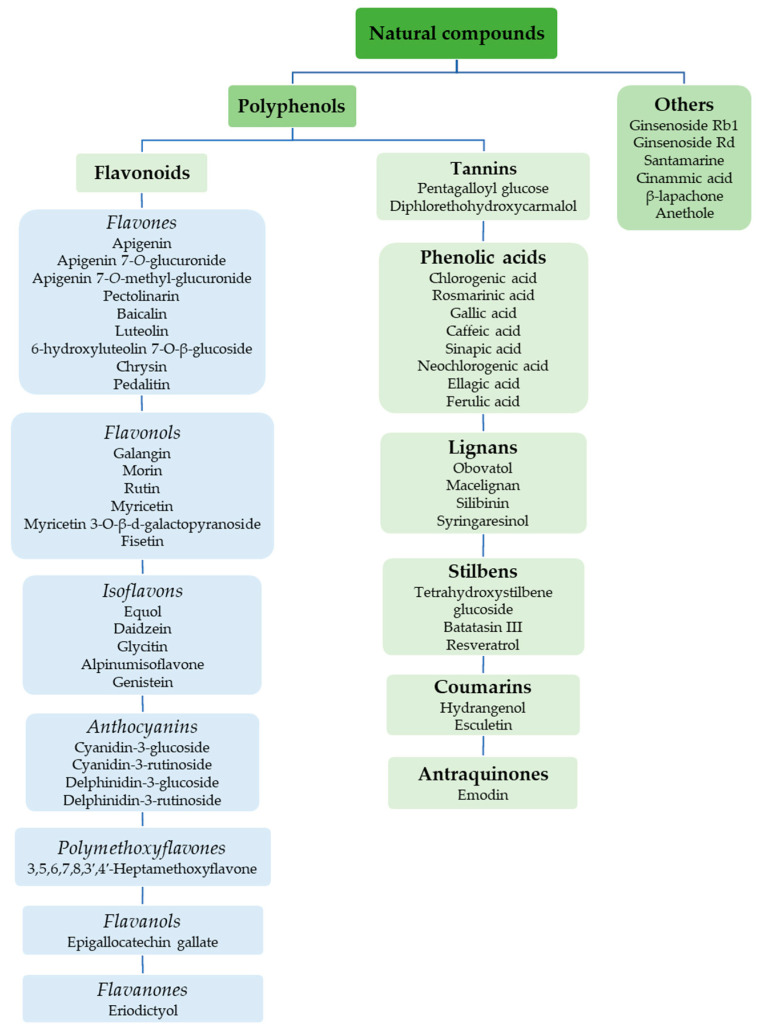
Classification of natural compounds with stimulating and protective effects on skin collagen type I.

Figure 1 shows natural compounds with stimulating and protective effects on skin type I collagen, which predominantly belong to polyphenols and have been classified into their main groups: flavonoids, phenolic acids, lignans, stilbenes, tannins, anthraquinones, and coumarins [47,49]. The largest group are flavonoids, whose structure contains the typical C6−C3−C6 flavonoid skeleton, and their classification is based on the structural differences. They include, among others, flavones, flavanols, flavanols, isoflavones, polymethoxyflavones, and anthocyanins. The presence of the phenolic hydroxyl group plays a key role in the capture of free radicals and depends on their number and position; but also, the double bond at the C2-C3 position is important to their antioxidant capacity [47,50]. In plants, flavonoids occur in free form as aglycones or in glycosylated form, forming O-glycosides or C-glycosides [47,49]. Several natural compounds, not classified as polyphenols, with a stimulating effect on skin collagen, have been marked as other (non-polyphenolic compounds) (Figure 1).

The structural formulas, effects, and mechanisms of action of natural compounds with a stimulating and protective effect on skin collagen type I are presented in Table 1. Most of the studies, the results of which are summarized in the table below, were conducted in vitro in human dermal fibroblasts (HDFs) and a few in vivo in mouse or rat models. They demonstrated both direct as well as protective effects on collagen against factors which most people are exposed to for most of their lives: UVA and UVB radiation, air pollution (fine dust particles (FDPs) and particulate matters (PMs)), hydrogen peroxide (H_2_O_2_), and the ingredients of cosmetic products (methylparaben (MP), propylparaben (PP), and benzophenone-3 (BP-3)).

### 3.1. Mechanisms of Action of Natural Compounds on Skin Collagen Type I

#### 3.1.1. TGF-β/Smad Pathway

TGF-β and especially the TGF-β/Smad pathway is a key regulator of collagen biosynthesis in human dermal fibroblasts [41,42]. This pathway also upregulates other important ECM components such as decorin (important in the regulation of fibrillogenesis), fibronectin, and versican, while it downregulates the expression of collagen-degrading enzymes (MMPs). This indicates that the TGF-β/Smad signaling pathway, by increasing the production of collagen and other ECM components and inhibiting ECM degradation, plays a very important role in maintaining the structural and mechanical integrity of the dermal ECM. Natural compounds such as apigenin [51], luteolin [54], galangin [58], myricetin-3-O-β-galactopyranoside [62], daidzein [65], glycitin [66], genistein [68], cyanidin- and delphinidin-3-glucosides and 3-rutinosides [69], 3,5,6,7,8,3′,4′-heptamethoxyflavone [70], eriodictoyl [71], chlorogenic acid [75], gallic acid [79], obovatol [85], macelignan [86], santamarine [96], and β-lapachone [98] stimulated the expression of collagen type I genes via the activation of TGF-β1, an increase in the phosphorylation of the Smad2/3, the expression of Smad4, and a decrease in the expression of Smad7 (Table 1). The TGF-β signaling pathway can be activated by connective tissue growth factor 2, which was stimulated at the mRNA and protein levels in HDFs in the presence of fisetin (3,7,3′,4′-tetrahydroxyflavone), and it was accompanied by the induction of procollagen and collagen type I gene expressions [63]. In addition to collagen type I, apigenin also stimulated the biosynthesis of collagen type III in human and mouse fibroblasts (NIH/3T3) via increased phosphorylation of the Smad2/3, and the results were confirmed in vivo in an aging mouse model [51]. After 1 month of administration of apigenin (5 μmol/L) to the dermis of mice, a significant increase in dermis thickness and collagen density were observed compared to the control mice [51]. Galangin (3,5,7-trihydroxyflavone) inhibited the expression of hsa-miR-4535 (which targets Smad4) and activated the Smad2/3/4 complex in human fibroblast cells exposed to both UVB and H_2_O_2_ [58]. The promotion of collagen biosynthesis signaling via the TGFβ/Smad pathway by galangin was also obtained in in vivo studies after the topical application of low (12 mg/kg) and high doses (24 mg/kg) of this compound to the dorsal skin of C57BL/6J nude mice [58]. Luteolin administered onto the bare dorsal skin of rats (60 mg/kg/day for 30 min) prevented against a UVB-induced decrease in the content of type I collagen via the activation of the TGF-β/Smad3 pathway [54]. The results of the stimulating effect of daidzein on the biosynthesis of type I collagen performed on newborn human skin fibroblasts were confirmed in a mouse model, where 200 μg/mL was used topically once a day for 6 weeks. This was accompanied by high levels of the phosphorylated Smad2/3 with no changes in their total content [65]. In Sprague Dawley rat models (SD rats), the subcutaneous administration of genistein at doses of 1 and 10 mg/kg daily for 12 weeks increased the expression of TGF-β1 as well as collagen thickness and skin breaking strength [68]. The upregulation of collagen gene expression may be mediated by the transcription factor (specificity protein 1 (Sp1)), which is induced by the activation of the TGF-β/Smad signaling pathway [93].

#### 3.1.2. MAPK Signaling Pathways

MAPK is a class of serine–threonine protein kinases including ERK, Jun N-terminal kinase (JNK), and p38 mitogen-activated protein kinase (p38), which play key roles in cell signaling [100]. ERK plays a special role in the regulation of collagen biosynthesis, while MAPK p38 plays a role in the degradation of ECM, including collagen [101]. After phosphorylation, MAPK proteins enter the nucleus and activate many transcription factors, such as activator protein 1 (AP-1), cellular myelocytomatosis oncogene (c-myc), cyclooxygenase-2 (COX-2), and nuclear factor-kappa B (NF-κB), which contribute to the increased activity of MMPs, collagen degradation, and accelerated skin aging [43,100,101]. Many plant-derived compounds, by acting on the different molecular targets of the MAPK signaling pathways, contribute to reducing these undesirable effects on collagen and skin.

##### The MAPK/ERK1/2 Pathway

Among the natural compounds targeting the ERK1/2 signaling pathway and stimulating collagen biosynthesis are the following: galangin [59,60], myricetin-3-O-β-galactopyranoside [62], alpinumisoflavone [67], 3,5,6,7,8,3′,4′-heptamethoxyflavone [70], eriodictoyl [71], epigallocatechin gallate [73], diphlorethohydroxycarmalol [74], rosmarinic acid [76], caffeic and sinapic acids [80], neochlorogenic acid [81], obovatol [85], macelignan [86], syringaresinol [88], hydrangenol [92], esculetin [93], emodin [94], and santamarine [96]. The vast majority of the listed compounds inhibited the phosphorylation of ERK1/2, while several of them (galangin, myricetin-3-O-β-galactopyranoside, syringaresinol, esculetin, emodin, and santamarine) had an activating effect on ERK1/2 kinase, and both of these effects were associated with a positive effect on type I collagen. For example, the treatment of UVA-exposed HDF with 10 µM of santamarine significantly increased ERK phosphorylation and the expression of collagen type I at both the mRNA and protein levels [96]. In fibroblasts treated with galangin at concentrations of 10 and 30 µM, there was an increase in the biosynthesis of collagen types I and III, which was also accompanied by an increase in ERK1/2 phosphorylation [59,60]. The treatment of the spontaneously transformed human keratinocyte cell culture (HaCaT keratinocytes) with myricetin-3-O-β-galactopyranoside (1, 5, and 25 µM) resulted in the suppression of all three MAPKs (ERK1/2, p38, and JNK), while in fibroblasts, an increase in the activation of ERK1/2 was observed [62]. The stimulating effects of syringaresinol (1, 5, and 25 µM) on collagen biosynthesis were also attributed to increased ERK activation in fibroblasts, whereas similarly to myricetin-3-O-β-galactopyranoside, syringaresinol inhibited the UV-induced phosphorylation of ERK1/2 in HaCaT keratinocytes [88]. Thus, the different results from the actions of these compounds on ERK1/2 (increase in the phosphorylation in fibroblasts and decrease in keratinocytes) may suggest a collagen production stimulatory mechanism mediated by this signaling pathway. However, not all studies have confirmed whether the activation of this signaling pathway is directly involved in the stimulation of collagen biosynthesis in cells exposed to natural compounds, alone or in combination with an appropriate stimulus (Table 1). For example, emodin at the concentration of 1 µM showed a stimulating effect on ERK1/2 kinase and 5′ AMP-activated protein kinase (AMPK) in HDFs [94]. To determine the involvement of these pathways (ERK and AMPK) in the upregulation of emodin-induced collagen expression, studies were performed in the presence of kinase inhibitors. The use of the ERK inhibitor (U0126) did not affect the expression of type I collagen, whereas it was inhibited by the AMPK inhibitor (compound c), suggesting that the regulatory enzyme for emodin-induced collagen expression was AMPK and not ERK1/2 [94]. Similarly, increased phosphorylation and the activation of AMPK were detected in the dorsal skin of BALB/C mice treated with resveratrol at a concentration of 100 µM, which stimulated the expression of type I collagen in HDFs and increased collagen fiber content in mice skin [91]. Esculetin at the concentrations of 10 and 100 µg/mL activated all three MAPK pathways (ERK1/2, p38, and JNK), which resulted in the stimulation of procollagen type I expression in HDFs [93]. Moreover, it was shown that esculetin increased Sp1 expression by activating these pathways, so Sp1 may be involved in the induction of procollagen type I expression by this compound via the activation of *COL1A1* transcription [93].

Other studies have also yielded conflicting results regarding the mechanism of ERK1/2-mediated collagen biosynthesis stimulation, reporting both the activation and inhibition of these kinases, which could be dependent on the cell type, culture conditions, and the type of stimulating factors [102,103].

##### The MAPK/p38/JNK/AP-1 Pathway

The MAPK/p38/JNK/AP-1 pathway is the main regulator of MMP expression, and the activation of this pathway leads to its excessive synthesis and collagen degradation [104,105]. MAPKs participate in the phosphorylation of c-Jun and c-Fos proteins. Phosphorylated JNK (pJNK) phosphorylates the c-Jun protein in the NH_2_-terminal domain, while phosphorylated ERK (pERK) activates the c-Fos at the COOH-terminal serine [106,107]. Phosphorylated c-Fos and c-Jun form the active AP-1 complex, which leads to increased MMP gene transcription [105,107]. It was shown that the treatment of skin fibroblasts with the following compounds—luteolin [54], myricetin 3-O-β-galactopyranoside [62], alpinumisoflavone [67], 3,5,6,7,8,3′,4′-heptamethoxyflavone [70], eriodictyol [71], gallic acid [79], caffeic and sinapic acid [80], neochlorogenic acid [81], macelignan [86], syringaresinol [88], hydrangenol [92], santamarine [96], and trans-cinnamic acid [97]—inhibited the phosphorylation of MAPKs (p38 and JNK) and consequently c-Fos and c-Jun, which was associated with a reduction in the expression of the main enzyme (MMP-1) responsible for the degradation of type I collagen (Table 1). In addition to MMP-1, other MMPs were inhibited in HDFs treated with natural compounds. For example, MMP-3 was downregulated by luteolin [54], obovatol [85], hydrangenol [92], santamarine [96], and trans-cinnamic acid [97], MMP-9 was downregulated by syringaresinol [88], while MMP-2, MMP-8, MMP-9, and MMP-13 were downregulated by epigallocatechin gallate [72] and diphlorethohydroxycarmalol [74], which was accompanied by the inhibition of p38 and JNK phosphorylation. The mechanism of the downregulation of MMP-1 and MMP-3 in HDFs via inhibiting the activation of the p38/JNK/AP-1 signaling pathway and the activation of the collagen-promoting TGF-β pathway by luteolin was confirmed in rats whose naked backs were coated with luteolin at a dose of 60 and 120 mg/kg/day and then exposed to UVB irradiation for 1 h every other day for 1 month [54].

##### The MAPK/NF-κB Pathway

The activation of this pathway and increased NF-κB binding to DNA are implicated in aging and the pathogenesis of many age-related disorders, because NF-κB is a critical transcription factor contributing to the senescence-associated secretory phenotype (SASP) including the production of pro-inflammatory cytokines, chemokines, and proteases [108,109]. The inhibition of the activation of NF-κB and the induction of inflammatory cytokines, such as TNF-α, COX-2, IL-1, IL-1β, and IL-6, which is taking place under the influence of UV as a result of the inflammatory response of the skin [13,16], is another mechanism of MMP inhibition and the promotion of collagen expression. Several polyphenolic compounds have been shown to inhibit the activation of this factor and related signaling pathways. Galangin at a concentration of 30 µM and alpinumisoflavone (25 and 50 µM) significantly inhibited the activation of NF-κB in skin fibroblasts, as well as the production of pro-inflammatory mediators such as IL-6, IL-1β, IL-8, and TNF-α [59,60,67]. Eriodictyol (2.5–40 µM), in addition to the above-mentioned pro-inflammatory factors, decreased the expression of COX-2 at the mRNA level and MMP-1 in fibroblasts [71]. Myricetin-3-O-β-galactopyranoside and syringaresinol showed protective effects against the UVA-induced upregulation of inflammatory factors (TNF-α, COX-2, and IL-1β) and MMPs (MMP-1, MMP-3, and MMP-9) [62,88]. The inhibition of the MAPK/NF-κB pathway, which resulted in a decrease in MMP-1 and an increase in collagen levels, was also observed in fibroblasts under the influence of caffeic and sinapic acids at a concentration of 100 µM each [80]. Apigenin, which has been shown to have a stimulating effect on collagen in HDFs and in mice [51], in other studies conducted in bleomycin-induced senescent human diploid foreskin fibroblasts (BJ) and in rats, strongly inhibited SASP expression. It was associated with the inhibition of NF-kB p65 activity via the IL-1 receptor-associated kinase (IRAK1)/the inhibitor of the NF-κB (IkBα) signaling pathway [110].

#### 3.1.3. The PI3K/Akt/mTOR Pathway

PI3K, through the production of phosphatidylinositol-3,4,5-triphosphate or phosphatidylinositol-3,4-bisphosphate, recruits Akt and phosphoinositide-dependent kinase (PDK1) to the plasma membrane, where PDK1 phosphorylates and activates Akt [111]. Once activated, Akt leaves the plasma membrane to phosphorylate intracellular substrates or transcription factors in the nucleus. Under the influence of galangin, glycitin, and esculetin, the level of phosphorylated Akt increased significantly, suggesting the contribution of the PI3K/Akt signaling pathway in collagen regulation in HDFs in addition to the MAPK pathways [59,66,93]. The activation of the PI3K/Akt pathway by TGF-β and IGF-1 thus increases the expression of type I procollagen [66,111,112].

Evidence for the mediation of this pathway in the stimulation of collagen type I biosynthesis by 20 μM of glycitin was the abolition of this effect in the presence of Akt/PI3K/mTOR inhibitors and the increase in the MMP-1 level [66]. Similarly, in the wound healing assay, glycitin showed a significant effect on fibroblast migration and no effect in the presence of the same inhibitors [66]. These data suggest that Akt/PI3K/mTOR, which was activated by TGFβ, may be the main signaling pathway mediating glycitin-induced collagen stimulation and cell migration.

#### 3.1.4. IGF-1/IGF-1R

IGF-1 is the primary growth factor responsible for inducing collagen biosynthesis in dermal fibroblasts [45,112]. IGF-1 signal transduction is mediated through the IGF-1R, which is a tyrosine kinase consisting of transmembrane glycoproteins α and β. Upon ligand binding, the receptor undergoes dimerization and activation and phosphorylates several substrates such as IRS-1 and IRS-2. Activated IRS proteins are recognized by the signaling molecules p85 and Grb2, which stimulate the Ras/Raf/MAPK and the PI3K/Akt/mTOR signaling cascades, mediating beyond the many collagen biosynthesis biological functions of IGF-1 including the regulation of proliferation, differentiation, and cell survival [45,46]. The increased expression of this growth factor and/or its receptors has been demonstrated under the influence of apigenin 7-*O*-glucuronide, apigenin 7-*O*-methyl-glucuronide, pectolinarin [52], and galangin [59,60] in skin fibroblasts and tetrahydroxystilbene glucoside in Kunming mice [89]. The inhibition of IGF-1R activation by the inhibitor AG1024 resulted in a decreased expression of collagen type I and III, indicating the involvement of the IGF-1 receptor in galangin-induced collagen biosynthesis in HS68 fibroblasts [60].

#### 3.1.5. The Nrf2/ARE Pathway

One of the main regulators of the expression of antioxidant proteins protecting cells from oxidative stress and oxidative damage is nuclear factor erythroid 2-related factor 2 (Nrf2) [113,114]. Nrf2 is negatively regulated by its inhibitor, Kelch-like ECH-associated protein 1 (Keap-1), whereas under the influence of oxidative stress, it is released from the Nrf2-Keap-1 complex and translocates to the nucleus, where it binds to the antioxidant response element (ARE) and induces the promoter region of antioxidant or detoxifying enzymes such as heme oxygenase 1 (HO-1), γ-glutamate-cysteine ligase (γ-GCLC), NAD(P)H quinone dehydrogenase 1 (NQO-1), glutamate cysteine ligase modifier subunit (GCLM), and glutamate cysteine ligase catalysis subunit (GCLC) [113,114]. UVA-induced Nrf2 deficiency resulted in an increased expression of collagen-degrading MMPs in skin fibroblasts [115]. In Hs68 cells transfected with small interfering RNA against Nrf2, the expression of Nrf2 and the enzymes HO-1, GSH, and NQO was reduced, which was accompanied by increased levels of collagenase and decreased levels of procollagen, confirming the important role of this pathway in protecting collagen from ROS [59]. The activation of Nrf2 and antioxidant enzymes has been reported in the presence of some polyphenolic compounds stimulating collagen biosynthesis. For example, galangin (3,5,7-trihydroxyflavone) caused increased levels of phosphorylation of Nrf2 and HO-1 in human dermal fibroblast HS68 treated with H_2_O_2_ [59]. Santamarin reversed the UVA-induced inhibition of the Nrf2-mediated antioxidant response, which resulted in the stimulation of Nrf2, SOD-1, and HO-1 expression at both mRNA and protein levels [96]. This was accompanied by a reduction in ROS, which has a destructive effect on cells and collagen, confirming the importance of this pathway in protection against cell aging. The anti-aging effects of trans-cinnamic acid were associated with an increased nuclear translocation of Nrf2 and the upregulation of Nrf2-dependent antioxidant genes, HO-1 and γ-GCLC, scavenging toxic ROS [97]. The results were confirmed by silencing Nrf2 with siRNA, resulting in enhanced ROS production and the inhibition of procollagen type I in UVA-irradiated cells [97]. In Nrf2 activation by trans-cinnamic acid in Hs68 cells, protein kinase C, AMPK, and casein kinase II signaling pathways were involved [97]. Another polyphenol that promoted the expression of mainly Nrf2 and HO-1 and to a lesser extent NQO-1, GCLM, and GCLC in mice with UVB-induced photoaging was hydrangenol, a natural dihydroisocoumarin [92].

#### 3.1.6. Other Mechanisms

The important regulators of MMP activity are intracellular inhibitors called the tissue inhibitors of metalloproteinases (TIMPs), of which TIMP-1 and TIMP-2 are the most studied [116,117]. An imbalance between high levels of MMPs and insufficient levels of TIMPs promotes the degradation of ECM components, including collagen; therefore, increasing the level of these inhibitors may be beneficial in preventing this process. For example, the use of 3 µM of myricetin increased the level of TIMP-1 and the ratio of this inhibitor to MMPs (MMP-1, MMP-2, and MMP-9), which contributed to the increase in procollagen type I and III in HDFs [61]. Similarly, after the treatment of 3D HDFs with 10 nM of equol, the increase in collagen type I and III concentrations was accompanied by an increase in TIMP-1 and a decrease in MMPs (MMP-1, MMP-3, and MMP-9) [64]. A decrease in the expression of type I collagen-degrading enzymes (MMP-1, MMP-2, or MMP-9) with an increase in TIMP-1 and/or TIMP-2 was also observed in daidzein-, eriodictyol-, rosmarinic acid-, and ferulic acid-treated skin fibroblasts [65,71,76,83].

Prolidase is a cytosolic imidodipeptidase that plays an important role in the recovery of proline from imidodipeptides, which are mainly derived from collagen degradation products, for collagen re-synthesis [118]. The activity of this enzyme is increased by the stimulation of the β1 integrin receptor [118]. In the skin fibroblasts of patients with osteogenesis imperfecta (OI) type I with an approximate 50% deficiency of type I collagen [119], an increase in prolidase activity and a normalization of the collagen level were demonstrated in the presence of apigenin 7-O-glucuronide, apigenin 7-O-methylglucuronide, and pectolinarin at a concentration of 30 µM [52]. The stimulation of type I collagen biosynthesis and the increase in prolidase activity by apigenin 7-O-glucuronide was accompanied by an increase in IGF-1R expression, whereas in the action of apigenin 7-O-methylglucuronide and pectolinarin, the β1-integrin-mediated pathway could be involved [52].

Important enzymes modifying collagen and determining its stable structure and proper secretion are collagen prolyl-4-hydroxylase (P4H) and β(1-O) galactosyltransferases (GLT25D1 and GLT25D2) [31,32,33,34]. The β subunit of the P4H enzyme is a PDI, which acts as a chaperone, preventing the aggregation of procollagen chains [34]. In addition, HSP47 protects the collagen triple-helix structure during the secretion of procollagen from the ER to the Golgi apparatus [34,35,36]. Under the influence of stimuli (BP-3, MP, and PP), changes in the expression of these enzymes and chaperones occurred in HDFs, while 100 µM of rosmarinic acid counteracted these changes [76,77].

**Figure 2:**
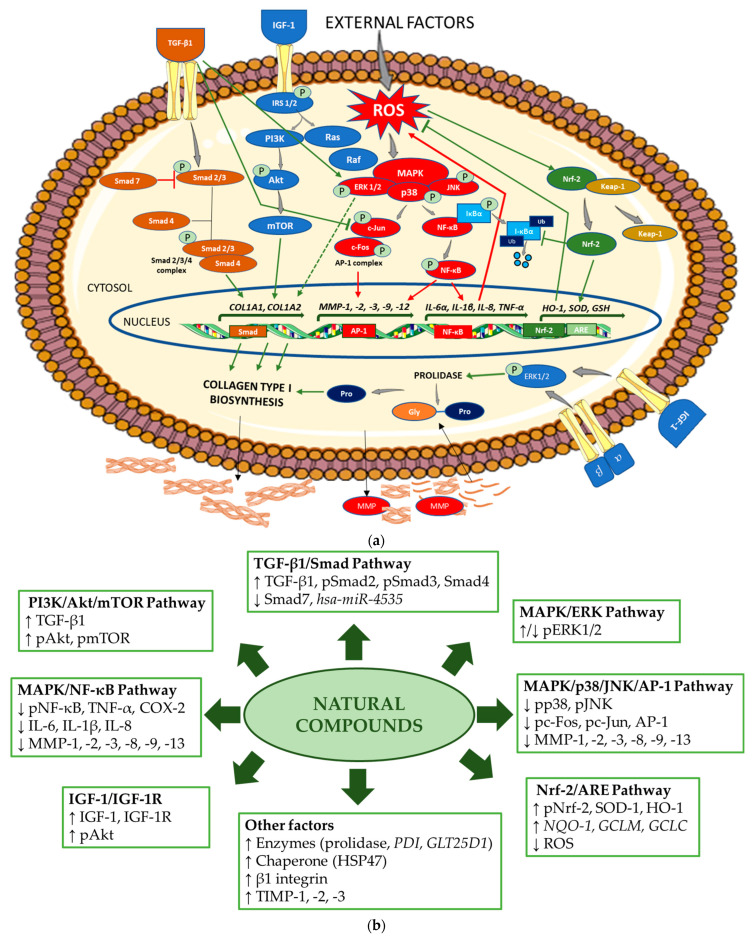
(**a**) Mechanism of collagen type I biosynthesis regulation. → stimulation, 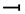 inhibition (in green indicates upregulation and in red, downregulation of collagen biosynthesis). Akt—protein kinase B; AP-1—activator protein 1; ARE—antioxidant response element; *COL1A1*—gene encoding the pro-alpha1 chains of type I collagen; *COL1A2*—gene encoding the pro-alpha2 chains of type I collagen; ERK—extracellular signal-regulated kinase; Gly—glycine; *GSH*—gene encoding glutathione; *HO-1*—gene encoding heme oxygenase 1; IGF-1—insulin-like growth factor 1; *IL*—gene encoding interleukin; IκBα—inhibitor of NF-κB; Keap-1—Kelch-like ECH-associated protein 1; MAPK—mitogen-activated protein kinase; MMP—matrix metalloproteinase; *MMP*—gene encoding matrix metalloproteinase; mTOR—mammalian target of rapamycin; NF-κB—nuclear factor-kappa B; Nrf-2—nuclear factor erythroid 2-related factor 2; PI3K—phosphoinositide 3-kinase; IRS-1/2—insulin receptor substrate 1/2; JNK—Jun N-terminal kinase; P—phosphorylated; Pro—proline; Smad—suppressor of mothers against decapentaplegic; Raf—rapidly accelerated fibrosarcoma; Ras—retrovirus-associated sequence; ROS—reactive oxygen species; *SOD*—gene encoding superoxide dismutase; TGF-β1—transforming growth factor beta 1; *TNF-α*—gene encoding tumor necrosis factor alpha; Ub—ubiquitin; α, β—subunits of integrin receptor. The figure was made using the templates available on the Servier Medical Art website (Creative Commons Attribution 4.0 unported license). (**b**) Mechanisms of action of the natural compounds on collagen type I. ↑—stimulation; ↓—inhibition. Akt—protein kinase B; AP-1—activator protein 1; ARE—antioxidant response element; COX-2—cyclooxygenase-2; ERK—extracellular signal-regulated kinase; GCLC—glutamate cysteine ligase catalysis subunit; GCLM—glutamate cysteine ligase modifier subunit; *GLT25D1*—gene encoding collagen galactosyltransferase 1; hsa-miR—homo sapiens microRNA; HO-1—heme oxygenase 1; HSP47—heat shock protein 47; IGF-1—insulin-like growth factor 1; IGF-1R—insulin-like growth factor 1 receptor; IL—interleukin 1; MAPK—mitogen-activated protein kinase; MMP—matrix metalloproteinase; NF-κB—nuclear factor-kappa B; NQO-1—NAD(P)H quinone oxidoreductase 1; Nrf-2—nuclear factor erythroid 2-related factor 2; pAkt—phosphorylated protein kinase B; pc-Fos—phosphorylated c-Fos protein; pc-Jun—phosphorylated c-Jun protein; *PDI*—gene encoding protein disulfide isomerase; pERK1/2—phosphorylated extracellular signal-regulated kinase 1/2; PI3K—phosphoinositide 3-kinase; pJNK—phosphorylated Jun N-terminal kinase; pmTOR—phosphorylated mammalian target of rapamycin; pNF-κB—phosphorylated nuclear factor-kappa B; pNrf-2—phosphorylated nuclear factor erythroid 2-related factor 2; pp38—phosphorylated p38 protein; pSmad—phosphorylated suppressor of mothers against decapentaplegic; ROS—reactive oxygen species; Smad—suppressor of mothers against decapentaplegic; SOD-1—superoxide dismutase 1; TGF-β1—transforming growth factor beta 1; TIMP—tissue inhibitor of metalloproteinase; TNF-α—tumor necrosis factor alpha.

Figure 2a schematically presents the mechanisms regulating collagen biosynthesis, while Figure 2b shows the mechanisms of the stimulating effects of the natural compounds on collagen biosynthesis and the inhibiting effects on the pathways, enhancing the degradation of this protein.

### 3.2. The Protective Effect of Natural Compounds Against the Unfavorable Influence of External Factors on Collagen Type I

#### 3.2.1. UV

The exposure of the general population to UV (UVA and UVB) radiation is impossible to avoid, and it is still increasing due to ozone layer depletion [15,16,19,20,120]. Although some doses of UV radiation are essential for the organism to synthetize vitamin D, an excessive amount of this radiation can be harmful to the human organism, especially the skin [120,121,122,123,124]. UVB radiation can penetrate the epidermis and reach the upper dermis, which leads to increased oxidative stress and causes erythema (sunburn), while UVA radiation can penetrate the dermis and reach the fibroblasts that synthesize collagen [120,121,122]. Exposure of the skin to UV radiation can cause some adaptive changes, including sclerosis, the degeneration of the fibrous tissues and blood vessels of the skin, and the acceleration of skin aging. Skin photoaging is indicated by an increased thickness of the epidermis, an irregularity in skin pigmentation, and damage to the skin ECM, which is caused by, among others, the UVA- and UVB-induced excessive production of ROS and pro-inflammatory cytokines, prostaglandins and leukotrienes, damage of DNA, and cell apoptosis [15,16,20,121,122,123]. The signaling pathways promoting collagen biosynthesis (TGFβ/Smad and MAPK/ERK) are disturbed, while the activated signaling pathways mediated by NF-κB and AP-1 promote collagen degradation [19,20,121,122,123]. The transcription factor NF-κB forms a complex with IκB, while the ROS-induced phosphorylation of IκB leads to its degradation and the activation of NF-κB [107,109]. NF-κB is translocated to the nucleus, where it causes an upregulation of pro-inflammatory factors (TNF-α, IL-1β, and IL-6) and MMPs. Similarly, AP-1 activation increases MMP transcription [105,106]. Further evidence of the adverse effects of UV on the skin is the damage to the epidermal barrier of the skin. Exposure of hairless mice to UVB radiation disrupts the epidermal barrier function, as evidenced by the increase in transepidermal water loss and the decrease in the expression of skin barrier-related markers such as involucrin, filaggrin, and aquaporin 3 (AQP3) [79,92]. In addition, UVB radiation negatively affected the content of HA, which is responsible for proper skin hydration [9,124]. The decrease in the expression of HA synthases and the increase in the expression of genes encoding hyaluronidases that degrade HA were demonstrated in UVB-irradiated hairless mice [92]. A reduction in collagen type I content and other important ECM components (e.g., HA and elastin), by inhibiting biosynthesis and increasing their degradation, accelerates skin photoaging, as was evidenced by a significant increase in the number of SA-β-Gal-positive senescent cells and the expression of senescent markers (p21 and p16) [53,59,60,91]. In mouse/rat models, along with collagen loss, skin aging was indicated through the appearance of wrinkles, rough texture, laxity, poor elasticity, dryness, and reduced skin resistance to stretching [54,58,79,92]. Sunburn and hyperpigmentation appearing as a result of skin exposure to UV radiation can lead to skin cancer [123,125]. The decrease in collagen type I expression due to UV exposure leading to negative effects on the skin was confirmed in studies of 270 human tissue samples [125]. The authors of these studies reported greater changes in the level and structure of collagen type I in photoaging compared to the physiologically aging skin [125].

**Figure 3:**
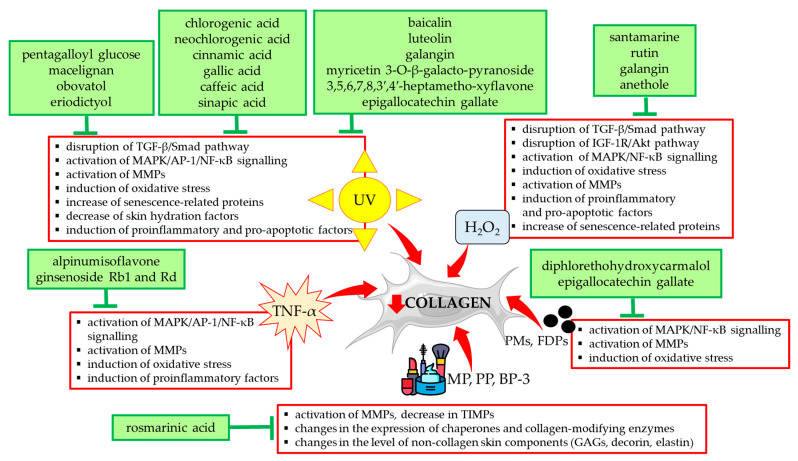
Natural compounds counteracting the negative effects of external factors on type I collagen in human skin fibroblasts. Akt—protein kinase B; AP-1—activator protein 1; BP-3—benzophenone-3; FDPs—fine dust particles; GAGs—glycosaminoglycans; H_2_O_2_—hydrogen peroxide; IGF-1R—insulin-like growth factor 1 receptor; MAPK—mitogen-activated protein kinase; MMPs—matrix metalloproteinases; MP—methylparaben; NF-κB—nuclear factor-kappa B; PMs—particulate matters; PP—propylparaben; Smad—suppressor of mothers against decapentaplegic; TGF-β—transforming growth factor beta; TIMPs—tissue inhibitors of metalloproteinases; TNF-α—tumor necrosis factor alpha; UV—ultraviolet. The figure was made using the templates available on the Servier Medical Art website (Creative Commons Attribution 4.0 unported license).

Other stimulants included in Table 1 such as H_2_O_2_ and TNF-α exerted a destructive effect on collagen type I in HDFs via the same mechanisms as UVA/UVB radiation. They are summarized in Figure 3 together with a presentation of natural compounds that counteracted these changes.

**Figure 4:**
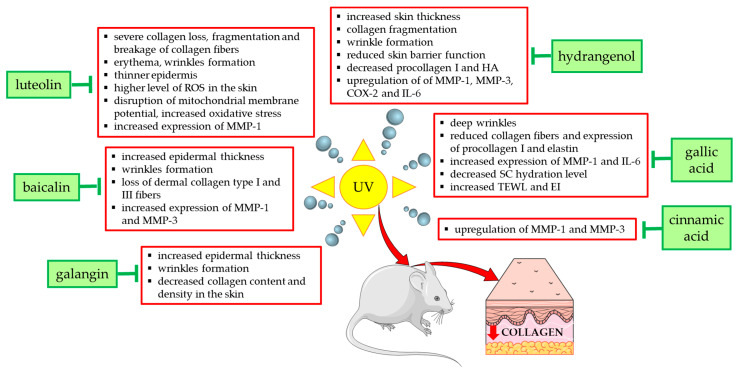
Natural compounds counteracting the negative effects of UVA/UVB radiation on type I collagen and skin in experimental mouse/rat models. COX-2—cyclooxygenase-2; EI—erythema index; HA—hyaluronic acid; IL-6—interleukin 6; MMP-1—matrix metalloproteinase 1; MMP-3—matrix metalloproteinase 3; ROS—reactive oxygen species; SC—stratum corneum; TEWL—transepidermal water loss; UV—ultraviolet. The figure was made using the templates available on the Servier Medical Art website (Creative Commons Attribution 4.0 unported license).

Figure 4 summarizes the effects and mechanisms of UVA/UVB radiation demonstrated in in vivo studies in mouse or rat models and shows the natural compounds that prevented these undesirable effects. For example, the topical application of low (12 mg/kg) and high doses (24 mg/kg) of galangin to the dorsal skin of C57BL/6J nude mice alleviated UVA/UVB radiation-induced skin damage and reduced epidermal hyperplasia, wrinkle formation, and skin aging [58]. Coating the bare dorsal skin of rats with luteolin (60 mg/kg/day for 30 min) before exposure to UVB radiation (1 h every other day for 1 month) increased the content of type I collagen and reduced skin damage (erythema and wrinkles) [54]. In reconstructed human skin tissue exposed to UVB radiation, an increased number and density of collagen fibers was observed after the topical application of resveratrol-enriched rice to the skin, which resulted in a reduction in wrinkles and skin inflammation [126].

#### 3.2.2. Air Pollution

Another factor that people around the world are exposed to is air pollution, and the skin, as the external barrier of the body, is in direct contact with it. The components of air pollution (PMs) are a mixture of microscopic particles and liquid droplets, which originate from artificial sources (combustion in mechanical and industrial processes, device emissions, open fires, and tobacco smoke) or natural sources (volcanoes, fires, sandstorms, and sea salt in aerosol) [127,128]. The components of PMs are organic chemicals, metals, dust particles, or soil, and in addition, they can carry compounds such as polycyclic aromatic hydrocarbons. In terms of size, particulate matters are divided into the following: PM 10 (smaller than 10 μm), PM 2.5 (smaller than 2.5 μm), and ultrafine particles (smaller than 0.1 μm) [127]. Due to their size, the particles can penetrate the skin, and they also enter the body through hair follicles and the respiratory system [129]. The correlation between the increase in the concentration of PMs in the air and premature skin aging or skin diseases is of increasing concern. They cause skin damage and diseases by inducing an inflammatory response and generating oxidative stress [128,129,130]. Moreover, PMs are carriers of polycyclic aromatic hydrocarbons and metals, which can additionally produce ROS and damage mitochondria [131]. An epidemiological study showed a direct relationship between exposure to PM and skin aging, as evidenced by the appearance of pigment spots and wrinkles [132]. It was reported that PM can induce the expression of MMP-1 and MMP-3 in skin fibroblasts and thereby increase the degradation of ECM proteins that maintain skin integrity, such as collagen type I [25,133]. The treatment of HDFs with PM showed an inhibition of collagen type I biosynthesis and an induction of MMP expression via the activation of MAPK, NF-κB, and AP-1 signaling pathways [73]. In the same study, it was shown that green tea epigallocatechin gallate (at concentrations of 12.5, 25, and 50 µM) significantly improved collagen biosynthesis and reduced the expression of MMPs (MMP-1, -2, -8, -9, and -13) as well as collagenase activity [73]. The same authors showed a beneficial effect of diphlorethohydroxycarmalol, an algal polyphenol (at concentrations of 25, 50, and 100 μM) on collagen type I in HDFs treated with PM (ERM-CZ-100) [74] via the same mechanism as epigallocatechin gallate [73] (Figure 3).

#### 3.2.3. Chemicals from Cosmetics Products

Parabens are among the most commonly used synthetic preservatives in cosmetics, medicines and food [22,134]. They are esters of 4-hydroxybenzoic acid, and the most frequently detected in cosmetics and accumulating in the human body in the highest concentrations are MP and PP [22]. Although parabens are considered safe in low concentrations, many scientific reports contradict this and provide evidence of the harmfulness of the excessive use of cosmetics containing these preservatives [22,134,135,136]. Parabens can be rapidly absorbed through the skin, and MP, despite having the lowest lipophilicity, has a greater ability to penetrate through the skin compared to other parabens and accumulates in an un-metabolized form in body tissues [22]. In addition, some factors, including UV, can enhance the absorption of parabens through the skin and their undesirable effects, including increased ROS generation [137]. Due to their estrogenic properties, they can contribute to the development of breast and skin cancer, obesity, and the risk of gestational diabetes [22,134,136]. In our previous studies, we have shown a negative effect of MP alone and in combination with PP on human skin fibroblasts and the biosynthesis of skin collagen [21,76]. Under their influence, at concentrations corresponding to human exposure, there was a decrease in cell proliferation, an increase in apoptosis, a decrease in total collagen biosynthesis, a decrease in gene expression encoding collagens type I, III, and VI, an increase in the activity of MMP-2, and a decrease in the activity of TIMPs (TIMP-1 and -2) [21,76]. Rosmarinic acid (at concentrations of 50, 100, or 150 μM) protected skin fibroblasts from the adverse effects of parabens [76].

In addition, rosmarinic acid prevented changes in HDFs exposed to one of the sun filters, BP-3 [77]. BP-3 (2-hydroxy-4-methoxybenzophenone) is one of the most commonly used chemical sunscreens, blocking both UVA and UVB radiation [23,138]. It is an ingredient not only in sunscreens but also in many cosmetic products used in skin care and personal hygiene, which most people use every day. Similarly to the parabens, there are scientific reports of BP-3 photo-carcinogenic, estrogenic, and anti-androgenic effects [23,139,140]. Similarly to parabens, BP-3 penetrates the skin and accumulates in the body [139,140,141]. Under the influence of BP-3, a decrease in the viability of HDFs, a decrease in the expression of type I collagen and PDI genes, and an increase in the expression of the GLT25D1 gene were demonstrated, which could suggest a disturbance in the hydroxylation and glycosylation of collagen [77]. In addition, the increased activity and expression of enzymes degrading ECM components (MMP-1, MMP-2, elastase, and hyaluronidase) as well as changes in the expression of the HSP47, decorin, HA synthases, sulfated glycosaminoglycans, and elastin were detected [77]. Figure 3 summarizes the negative effects of parabens and BP-3 on HDFs, which were significantly reduced or normalized by rosmarinic acid at a concentration of 100 µM.

### 3.3. Examples of Clinical Studies on the Use of Collagen Type I Stimulating Natural Compounds

In a clinical study, it was assessed that the use of a cream (2 g in the morning and evening for 4 weeks) with the addition of rutin (2% by weight) by 40 women aged 30–50 increased the thickness of the dermis (by improving the density of the dermis), improved skin elasticity, and also had an anti-wrinkle effect, including a beneficial effect on wrinkles under the eyes. Importantly, no adverse effects of the cream were observed in the study group [57]. In another study, the effect of a peel containing 14% ferulic acid (eight chemical peels at 1-week intervals) was assessed in 25 women aged 45–60 with signs of photoaging. A skin assessment was performed between 8 and 12 weeks after the first treatment and showed statistically significant improvement in skin hydration, topography, the melanin level, and the severity of erythema [142]. In addition, ferulic acid has been used as a stabilizer or adjuvant in various cosmetic formulations containing vitamins (C and E), acids (azelaic, mandelic, and phytic), or resorcinol, which resulted in its more effective action on the skin compared to the action of the ferulic acid alone. The details of the clinical trials using ferulic acid in combination with other compounds and the application of modern technologies to increase its bioavailability have been described in a review by Cavalcanti et al. [143]. In a study conducted on a group of 20 volunteers, each participant applied a cream containing resveratrol to their face in the morning and evening for 6 weeks [144]. The tested cream improved the functioning of the skin’s hydrolipid barrier and increased its hydration, improving the overall condition of the skin without showing any allergic effects. A study conducted on 30 subjects aimed to assess the effect of a liotropic liquid crystal emulsion containing resveratrol on the penetration capacity, the presence of skin pores, and pigmentation levels [145]. After 45 days of using this emulsion, signs of pore involution were shown on the forehead, but there was no improvement in the other parameters. However, volunteers reported an increase in skin hydration and a reduction in skin oiliness. In total, 54% of volunteers noted an improvement in the skin condition after only seven applications of this product, and 25% of the volunteers noted some reduction in wrinkles [145]. After 30 days of daily use of resveratrol in combination with β-cyclodextrin as an excipient, a visible improvement in the skin condition was observed in eight women aged 45 to 70 years with symptoms of photoaging [146].

In a clinical study conducted on 55 women (aged 40–60) who applied resveratrol preparations in combination with baicalin and vitamin E to the face and neck every night for 12 weeks, an improvement in skin elasticity and thickness was demonstrated [147]. Clinical evaluation showed a statistically significant reduction in fine lines and wrinkles, increased skin firmness and elasticity, and decreased skin roughness and hyperpigmentation. In the periorbital area, an 18.9% improvement in dermis thickness was demonstrated. In addition, a trend toward increased collagen type I expression was observed [147]. Effective protection against sunburn was demonstrated in a study conducted on 15 volunteers by a stable derivative of resveratrol (resveratrate) [148]. A group of 20 women applied a cream containing 0.8% resveratryl triacetate twice a day to their face for 4 and 8 weeks, which resulted in a reduction in the area and volume of wrinkles and sagging, as well as an increase in skin elasticity, density, hydration, and brightness [149]. The use of an emulsion containing 2% trans-resveratrol by a group of 20 people for a period of 8 weeks contributed to increased skin elasticity and density and diminished its roughness and dispensability [150].

In a study of 30 women aged 45–55 who applied gels containing estrogen or genistein to their faces twice a day, an increase in the amount of collagen types I and III in the face skin was demonstrated after 24 weeks, but the effect of genistein was weaker compared to estrogen [151]. Epigallocatechin-3-gallate was applicated topically on the skin with created scars in 62 healthy people. The effect was a reduction in the thickness of the scar (after 1–3 weeks), as well as an increase in its hydration (week 3) and elasticity (week 4) [152].

The topical administration of 0.5% equol racemate improved the condition of the skin in a study including 64 women (aged between 40 and 60 years). It was applicated in the form of an emulsion with powder equol, as well as microencapsulated equol for 8 weeks, two times a day. In both cases, a reduction in skin roughness and an improvement in skin texture and smoothness were observed. An additional beneficial effect of this compound was the lengthening of the telomeres [153].

It is also important to highlight the limitations of these studies, which are related to the lack of standardization regarding the dosage of natural compounds, their forms and delivery systems, the duration of the studies, and the methods of assessing their effects and safety, which would increase the comparability and reliability of the results. An analysis of these studies using the Cochrane Risk of Bias tool indicated that some of them may raise concerns because information on the order of allocation [57] or randomization [144] was missing. The studies included too-small groups of participants [145,146,151,152,153] or used a short study duration [145,152] and also did not include different ethnic groups.

Although natural compounds are generally considered safe and no adverse effects were noted in most of the studies [57,142,144,146,147,149,151], undesirable effects of their use have also been reported. One participant dropped out of a study on the efficacy of resveratrol due to increased skin oiliness after using an emulsion containing this compound [145], while in another study, one person reported the appearance of pimples after 4 weeks of using an emulsion containing 2% resveratrol [150]. One study also raised the issue of possible side effects during the long-term use of natural cosmetics on large body surfaces [151]. According to some researchers, natural compounds used at higher doses can have pro-oxidant and mutagenic effects, may affect drug metabolism, the activity of important metabolizing enzymes, and thyroid and reproductive functions, or disrupt intestinal flora [154,155]. Therefore, it is extremely important to develop effective but also safe doses, especially with regard to long-term safety. Some authors also pointed out that in their study based on patient self-control, it was difficult to assess whether the beneficial effects of the cream were related to the action of the added polyphenol or other ingredients due to the lack of appropriate controls [149]. In connection with the above, it is very important to understand the effects of natural compounds used in combination with other natural or synthetic ingredients contained in cosmetic products. Their administration with vitamins, other antioxidants, or bioactive substances may produce synergistic effects that may enhance their beneficial effects on the skin, but additional research is needed to understand these complex interactions and design optimal formulations. A major challenge in the topical application of natural compounds to the skin is their low bioavailability and limited penetration into the target site, which is the dermis. However, the use of innovative encapsulation methods can improve their efficacy, effectiveness, and safety in skin care and anti-aging applications.

## 4. Conclusions

As the average life expectancy increases, maintaining a healthy skin appearance by preventing signs of aging has become a very important social aspect, which is supported by the dynamically developing pharmaceutical and cosmetic industry, and, in particular, products based on natural ingredients. Therefore, scientific research aimed at assessing the therapeutic potential of natural compounds and the safety of their application to the skin is of great importance. It should be emphasized that the natural compounds stimulating the biosynthesis of type I collagen have many other properties that are beneficial to the skin, such as reducing oxidative stress and inflammation and improving the skin barrier and hydration, which additionally makes them attractive therapeutic agents. Their high therapeutic potential has been confirmed in in vivo studies on mouse and rat models and on volunteers, in whom a significant improvement in the skin condition was achieved. It is also worth mentioning the limitations of this review. Firstly, it does not include plant extracts with a stimulating effect on skin type I collagen, and secondly, it provides only examples of clinical studies. When analyzing these studies, it is necessary to emphasize the need for standardization and increasing the number of long-term clinical trials, including larger and different ethnic groups, allowing for the effective and safe application of these compounds to the skin.

## Figures and Tables

**Table 1:** Structural formulas, effects, and mechanisms of action of natural compounds stimulating and protecting skin collagen type I in human dermal fibroblasts or mouse/rat models; the genes encoding the corresponding proteins are given in italics.

Name of Compound/ Structural Formula	Research Model (Concentration)	Stimuli (Intensity)	Effects/Mechanisms of Action	Ref.
FLAVONOIDS				
Flavones				
Apigenin 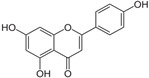	HDFs (0.1–10 µM) C57BL/6 mice (5 µM)	None	↑ *COL1A2, COL3A1* ↑ pSmad2, pSmad3 (-) MMP-1, MMP-2, MMP-9, TIMP-1 (-) pJNK, pERK, p38 ↑ collagen density, dermal thickness	[51]
Apigenin 7-*O*-glucuronide ** 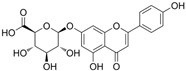 ** Apigenin 7-*O*-methyl- glucuronide ** 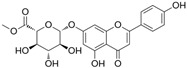 ** Pectolinarin 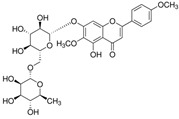	HDFs OI (30 µM)	None	↑ total collagen synthesis (assay with 5-[^3^H]proline) ↑ secretion of collagen ↑ prolidase activity, IGF-1R, β1 integrin (-) MMP-2, MMP-9, MMP-3	[52]
Baicalin 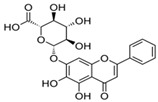	C57BL/6 mice (0.5 and 1 mg/cm^2^ skin area) HDFs (6.25–25 µg/mL)	UVB (6.9 J/cm^2^) UVB (10 mJ/cm^2^)	↑ *COL1A1, COL3A1* ↑ procollagen type I and III in the skin ↓ *MMP-1, MMP-3*, MMP-1, MMP-3 ↓ epidermal thickness ↓ premature senescence ↓ p53, p21, p16, γ-H2AX	[53]
Luteolin 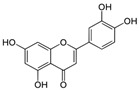	HDFs (10, 20, 40 µM) SD rats (60 and 120 mg/kg)	UVB (20 mJ/cm^2^) UVB (300 mJ/cm^2^)	↑ collagen type I ↑ TGF-β, Smad3, SIRT3, SOD, ROS ↓ p38, pJNK, c-Jun, MMP-1, MMP-3 ↑ collagen type I ↑ TGF-β, Smad3, SIRT3, SOD, ROS ↓ p38, pJNK, c-Jun, MMP-1, MMP-3 ↓ skin damage, erythema, wrinkles	[54]
6-hydroxyluteolin 7-O-β- glucoside 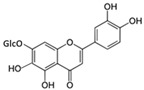 Pedalitin 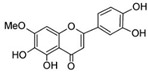	HDFs (1, 2, 40 μM)	None	↑ *COL1A1*, *COL1A2, COL3A1* ↑ total soluble collagen ↑ *HSP47* ↓ *MMP-2*	[55]
Chrysin 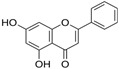	HDFs (20, 40, 80 µM)	None	↑ total collagen (hydroxyproline content)	[56]
Flavonols				
Morin 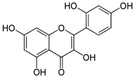	HDFs (80 µM)	None	↑ total collagen (hydroxyproline content)	[56]
Rutin 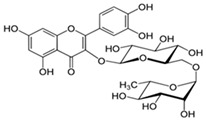	HDFs (80 µM)	None	↑ total collagen (hydroxyproline content)	[56]
	HDFs (1, 10, 50 µM)	H_2_O_2_ (0.2 mM)	↑ *COL1A1* ↓ *MMP-1* ↓ ROS	[57]
Galangin 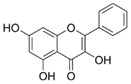	HDF HS68 (30 μM) C57BL/6J mice (12 and 24 mg/kg)	H_2_O_2_ (0.2 mM) UVB (40 mJ/cm^2^) UVB (150 mJ/cm^2^)	↑ collagen type I and III ↑ TGF-β, Smad4, p-Smad2/3 ↓ *hsa-miR-4535*, MMP-1 ↑ collagen content and density ↓ wrinkle formation ↑ TGF-β, Smad4, pSmad2/3 ↓ *hsa-miR-4535,* MMP1	[58]
	HDF Hs68 (10 and 30 µM)	H_2_O_2_ (0.2 mM)	↑ collagen type I and III ↑ IGF1-R, pAkt, pERK, pNrf2, HO-1, Bcl-xL ↓ pNF-κB, IL-6, IL-1β, TNF-α ↓ p65, p53, p16, p21	[59]
	HDF Hs68 (30 μM)	None H_2_O_2_ (0.2 mM)	↑ collagen type I and III ↑ IGF-1R, p-ERK ↑ collagen type I and III ↑ IGF-1R, p-ERK ↓ pNF-κB, IL-1β, IL-6, TNF-α, MMP-1 ↓ SA-β-gal, p53, p21, p16	[60]
Myricetin 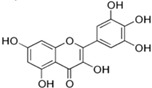	HDFs (3 μM)	None	↑ procollagen type I and III ↓ *MMP-1, MMP-2, MMP-9* ↑ TIMP-1, TIMP1/MMP ratio	[61]
Myricetin 3-O-β-galacto- pyranoside 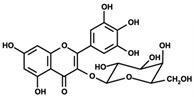	HDFs (1, 5, 25 μM)	UVA (19 J/cm^2^)	↑ collagen type I ↑ TGF-β, pSmad2/3, Smad4, pERK ↓ MMP-1, MMP-3, MMP-9 ↓ COX-2, iNOS, TNF-α, IL-1β ↓ pp38, pJNK, pc-Fos, pc-Jun	[62]
Fisetin ** 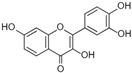 **	HDFs (10, 25, 50 µM)	None	↑ *COL1A2* ↑ *CCN2, Smad 2* ↑ CCN2, TGF-β1, TGF-β2, TGF-β3	[63]
Isoflavons				
Equol 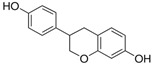	HDFs and 3D HDFs (10 nM) Human skin barrier equivalents (EFT cultures) (1.2%)	None	↑ collagen type I C-terminal propeptide ↑ collagen type I and III ↓ MMP-1, MMP-3 ↑ *COL1A1*, *TIMP-1* ↓ MMP-1, MMP-3, MMP-9	[64]
Daidzein 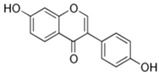	nHDFs (0.5, 5, 50 μg/mL) BALB/C mice (200 μg/mL)	None	↑ *COL1A1* ↑ *TGF-β*, p-Smad2/3, *TIMP-1* ↓ *MMP-1, MMP-2* ↑ *COL1A1,* collagen type I ↑ *TGF-β* ↓ *MMP-1, MMP-2*	[65]
Glycitin 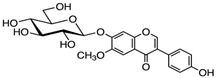	HDFs (20 µM)	None	↑ *COL1A1, COL3A1,* collagen type I and III ↑ *TGFβ-1*, TGFβ-1, pAKT, pmTOR, MMP-2 ↓ MMP-1	[66]
Alpinumisoflavone 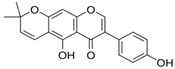	HDFs (25 and 50 µM)	TNF-α (20 ng/mL)	↑ *COL1A1* ↓ *MMP-1*, MMP-1, ROS, iNOS, COX-2 ↓ *IL-1β, IL-6, IL-8,* IL-1β, IL-6, IL-8, ↓ NF-κB (p65), AP-1 ↓ pERK, pJNK, pp38	[67]
Genistein 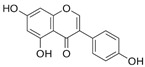	SD rats (1 and 10 mg/kg) daily, for 12 weeks, subcutaneous administration	None	↑ collagen thickness ↑ TGF-β1, MMP-2, MMP-9 ↑ TIMP-1, TIMP-2 ↑ skin breaking strength	[68]
Anthocyanins				
Cyanidin-3-glucoside (C3G) ** 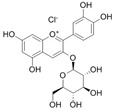 ** Cyanidin-3-rutinoside (C3R) 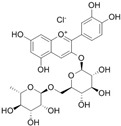 Delphinidin-3-glucoside (D3G) 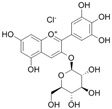 Delphinidin-3-rutinoside (D3R) 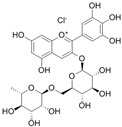	HDFs TIG113 (C3G, C3R, D3G and D3R—10 µM) BCE (1–10 µg/mL) OVX SD rats (BCE 3%—dietary administration)	None	↑ *COL1A1, COL3A1, TIMP-3* ↑ collagen type I and III ↑ TGF-β, IGF-2, IGFBP2, IGFBP5 ↑ collagen thickness	[69]
Polymethoxyflavones				
3,5,6,7,8,3′,4′-Heptametho- xyflavone 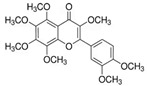	nHDFs (50, 100, 200 μg/mL)	UVB (20 mJ/cm^2^)	↑ procollagen type I C-peptide ↑ Smad3 ↓ Smad7, MMP1, pERK, pJNK, pc-Jun, c-Fos (-) pp38	[70]
Flavanones				
Eriodictyol ** 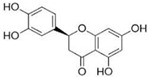 **	HDFs FEK-4 (2.5, 5, 10, 20, 40 µM)	UVA (150 KJ/m^2^)	↑ *COL1A1* ↓ MMP-1, ROS, pp38, pERK, pJNK ↓ *IL-1β, IL-6, TNFα*, *TGFβ*, COX-2, *NF-κB* ↑ TIMP-1, SOD	[71]
Flavanols				
Epigallocatechin gallate 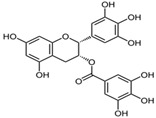	HDFs (10 µg/mL)	UVA (10 mW/cm^2^)	↑ total collagen (soluble and matrix)	[72]
	HDFs (12.5, 25, 50 µM)	FDPs (ERM-CZ100) (200 µg/mL)	↑ procollagen content ↓ MMP-1, MMP-2, MMP-8, MMP-9, MMP-13 ↓ collagenase activity ↓ ROS, p50, p65, pc-Jun, pERK, pJNK, pp38	[73]
TANNINS				
Pentagalloyl glucose 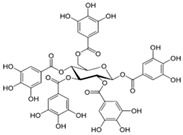	HDFs (10 µg/mL)	UVA (10 mW/cm^2^)	↑ total collagen (soluble and matrix)	[72]
Diphlorethohydroxycarmalol ** 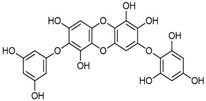 **	HDFs (25, 50, 100 µM)	PMs (ERM-CZ100) (200 µg/mL)	↑ collagen content ↓ MMP-1, MMP-2, MMP-8, MMP-9, MMP-13 ↓ collagenase activity ↓ ROS, p50, p65, pc-Jun, pERK, pJNK, pp38	[74]
PHENOLIC ACIDS				
Chlorogenic acid 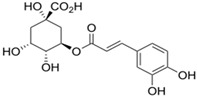	eHDFs CCC-ESF-1 (0.1–10 µM) HDFα (3 and 30 µM)	None UVA (12 J/cm^2^)	↑ *COL1A2*, collagen type I ↑ collagen type I secretion ↑ *COL1A1*, collagen type I ↑ p-Smad2/3, Rad51 ↓ MMP-3, ROS, C-PARP, γ-H2AX (-) COL1A2, *COL3A1, COL5A1, MMP-1, MMP-3* (-) pERK	[75]
Rosmarinic acid 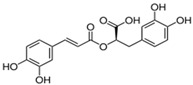	HDFs CRL-1474 (50–150 μM)	MP and PP (0.001% and 0.0003%; 0.003% and 0.001%; 0.01% and 0.003%)	↑ *COL1A1*, *COL1A2, COL3A1, HSP47* ↑ collagen type I, HSP47 ↓ *MMP-1, MMP-2, MT1-MMP* ↓ MMP-1, MMP-2, MT1-MMP ↑ *TIMP-1, TIMP-2* ↓ pERK1/2	[76]
	HDFs CRL-1474 (100 μM)	BP-3 (0.1–100 µM)	↑ *COL1A1*, *PDI, GLT25D1* ↑ collagen type I ↓ *MMP-1, MMP-2,* MMP-1, MMP-2	[77]
	HDFs OI (0.1, 1, 10 µM)	None	↑ *COL1A1* ↑ procollagen type I, collagen type I ↓ *MMP-2, MMP-9*	[78]
Gallic acid 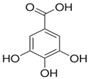	HDFs (1 and 10 µM) HDFs (1 and 10 µM) SKH-1 mice (1 and 5%)	None UVB (144 mJ/cm^2^) UVB (100–200 mJ/cm^2^)	↑ procollagen type I ↓ pc-Fos, pc-Jun, MMP-1, IL-6 ↑ procollagen type I ↓ pc-Fos, pc-Jun, MMP-1, IL-6 ↑ procollagen type I, TGF-β1 ↓ ROS, MMP-1, IL-6 ↓ wrinkle formation	[79]
Caffeic acid 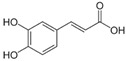 Sinapic acid 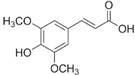	HDF Hs68 (100 µM)	UVB (30 mJ/cm^2^)	↑ total soluble collagen ↓ MMP-1, pERK, pJNK, pp38, NF-_K_B p50, ROS	[80]
Neochlorogenic acid 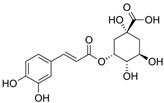	HDF Hs68 (50, 100, 200 μM)	UVB (15 mJ/cm^2^)	↑ procollagen type I ↓ MMP-1, pp38, pERK, pc-Fos, pc-Jun (-) pJNK	[81]
Ellagic acid 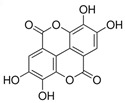	HDFs (2 µg/mL)	None	↑ *COL1A1* ↑ collagen deposition in the ECM	[82]
Ferulic acid 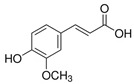	HDF CCD-986sk (5, 10, 20 µg/mL)	UVB (20 mJ/cm^2^)	↑ procollagen type I ↑ TIMP-1 ↓ MMP-1	[83]
	HDFs (10 and 20 µM)	UVA (10 J/cm^2^)	↑ *SOD, CAT* ↓ *MMP-1, MMP-3, p16* ↓ ROS ↓ % of senescent cells	[84]
LIGNANS				
Obovatol ** 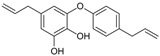 **	HDFs (1, 2.5, 5 μM)	UVB (40 mJ/cm^2^)	↑ procollagen type I ↑ TGF-β, Smad3 ↓ MMP-3, Smad7, AP-1, c-Fos, c-Jun ↓ pERK, pJNK, pp38	[85]
Macelignan 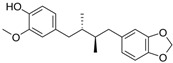	HDF Hs68 (1, 5, 10 μM)	UVB (20 mJ/cm^2^)	↑ *COL1A1,* procollagen type I C-peptide ↑ Smad3 ↓ Smad7, MMP1, *MMP-1*, ROS ↓ pERK, pJNK, pp38, pc-Jun (-) c-Fos	[86]
Silibinin ** 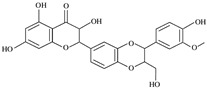 **	Wistar rats with skin wound (10% and 20% (*w*/*v*) powder)	None	↑ collagen (hydroxyproline content) ↑ *MMP-3* ↑ closure of wounded skin	[87]
Syringaresinol ** 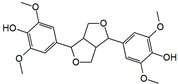 **	HDFs (1, 5, 20 µM)	UVA (10 J/cm^2^)	↑ procollagen type I, pERK ↓ MMP-1, MMP-9, TNF-α, COX-2, IL-1β ↓ JNK, pc-Fos, pc-Jun	[88]
STILBENS				
Tetrahydroxystilbene glucoside 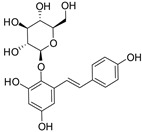	Kunming mice (180 mg/kg, gastric lavage)	None	↑ collagen content ↑ thickness of dermal layer ↓ IGF-1, IGF-1R	[89]
Batatasin III ** 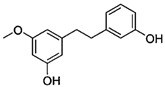 **	HDFs (3.182 μg/mL)		↑ procollagen	[90]
Resveratrol ** 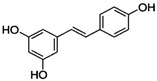 **	HDFs (100 μM) BALB/C mice (100 μM)	UVA (16 J/cm^2^) UVA (0.35 J/cm^2^, with increase of 5% per day)	↑ collagen type I ↓ MMP-1, SA-β-gal, p21, ROS ↑ collagen fiber content ↑ pAMPK ↓ MMP-1, p21 ↓ epidermal layer thickness	[91]
COUMARINS				
Hydrangenol ** 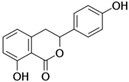 **	HR-1 hairless mice (5, 10, 20, 40 mg/kg), oral administration	UVB (60–240 mJ/cm^2^, stepwise increased irradiation)	↑ *COL1A1*, procollagen type I ↑ Nrf2, HO-1, *NQO-1, GCLM, GCLC* ↓ MMP-1, MMP-3, COX-2, IL-6, pSTAT1 ↓ pp38, pERK, pc-Fos, pc-Jun ↓ wrinkle formation, dermis thickness	[92]
Esculetin 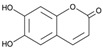	HDFs (10 and 100 µg/mL)		↑ *COL1A1,* procollagen type I ↑ pERK, pp38, pJNK ↑ pAkt ↑ Sp1	[93]
ANTHRAQUINONES				
Emodin 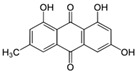	HDFs Hs27 (0.01–1 μM)	None	↑ *COL1A1, COL1A2,* collagen type I ↑ pAMPK, pERK1/2 (-) *COL3A1, COL5A1* (-) pFAK, pp38, pSMAD2	[94]
OTHERS (NON-POLYPHENOLIC COMPOUNDS)				
Ginsenoside Rb1 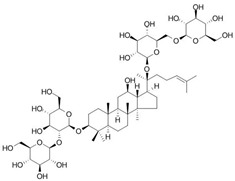 Ginsenoside Rd 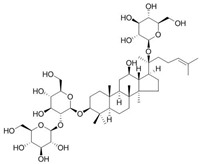	HDFs (50 and 100 μM)	TNF-α (20 μg/mL)	↑ collagen type I in medium (-) MMP-1	[95]
Santamarine 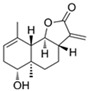	HDFs (1, 5, 10 μM)	UVA (8 J/cm^2^) H_2_O_2_ (500 µM)	↑ *COL1A1*, procollagen type I ↑ TGF-β, pSmad2/3, Smad4, pERK, ↑ *Nrf2, SOD-1, HO-1,* Nrf2, SOD-1, HO-1 ↓ *MMP-1*, MMP-1, MMP-3, MMP-9 ↓ pp38, pJNK, pc-Fos, pc-Jun, Smad7 ↓ ROS	[96]
*Trans*-Cinnamic acid 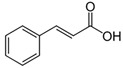	HDF Hs68 (20, 60, 100 μM) HDF Hs68 (20, 60, 100 μM) BALB/c-nu mice (20 and 100 mM)	UVA (3 J/cm^2^) None UVA (3 J/cm^2^)	↑ procollagen type I ↓ MMP-1, MMP-3, ROS, pc-Fos ↑ Nrf2, HO-1 and γ-GCLC ↑ procollagen type I ↓ MMP-1, MMP-3	[97]
Β-lapachone ** 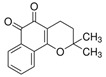 **	HDFs (0.01–0.1 μg/mL)	None	↑ *COL1A1*, procollagen type I C-peptide ↑ collagen type I ↑ p-Smad2/3	[98]
Anethole 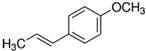	HDFs (1 μM)	None	↑ *COL1A1*, total collagen ↓ MMP-2	[99]

↑—stimulation; ↓—inhibition; (-) no effect. AMPK—adenosine monophosphate-activated protein kinase; AP-1—activator protein 1; BCE—blackcurrant extract; CAT—catalase; CCN—cellular communication network factor; *COL1A1*—gene encoding the pro-alpha1 chains of type I collagen; *COL1A2*—gene encoding the pro-alpha2 chains of type I collagen; COX-2—cyclooxygenase-2; C-PARP—poly(ADP-ribose) polymerase-1 cleavage; eHDFs—embryonic human dermal fibroblasts; FAK—focal adhesion kinase; FDPs—fine dust particles; GCLC—glutamate-cysteine ligase catalytic; GCLM—glutamate-cysteine ligase modifier; GLT25D1—glycosyltransferase 25 domain 1; H_2_O_2_—hydrogen peroxide; HDFs OI—human dermal fibroblasts obtained from osteogenesis imperfecta patients; HDFs—human dermal fibroblasts; HDFα—adult human dermal fibroblast, HO-1—heme oxygenase 1; hsa-miR—homo sapiens microRNA; HSP47—heat shock protein 47; IGF-1—insulin-like growth factor 1; IGF-1R—insulin-like growth factor 1 receptor; IL—interleukin; iNOS—inducible nitric oxide synthase; JNK—c-jun N-terminal kinase; MMP—matrix metalloproteinase; MP—methylparaben; MT1-MMP—membrane-type 1 matrix metalloproteinase; mTOR—mammalian target of rapamycin; NF-κB—nuclear factor kappa B; nHDFs—newborn human skin fibroblasts; NQO-1—quinone oxidoreductase 1; Nrf-2—nuclear factor erythroid 2-related factor 2; OVX—ovariectomized; pAkt—phosphorylated protein kinase; PDI—protein disulfide isomerase; pERK—phosphorylated extracellular signal-regulated kinase; PMs—particulate matters; pNF-κB—phosphorylated nuclear factor kappa B; PP—propylparaben; ROS—reactive oxygen species; SD—Sprague Dawley; SMAD—suppressor of mothers against decapentaplegic; SOD—superoxide dismutase; SP1—specificity protein 1; STAT—signal transduction and activation of transcription; TGF-β—transforming growth factor β; TIMP—tissue inhibitor of metalloproteinase; TNF-α—tumor necrosis factor-alpha; UV—ultraviolet; γ-H2AX—gamma histone family member X.

## Data Availability

All of the data are contained within the article.

## Abbreviations

Akt—protein kinase B; AMPK—5′ AMP-activated protein kinase; AP-1—activator protein 1; AQP3—aquaporin 3; ARE—antioxidant response element; BCE—blackcurrant extract; BJ—human diploid foreskin fibroblasts; BP-3—benzophenone-3; CAT—catalase; CCN— cellular communication network factor; c-Jun—transcription factor Jun; c-myc—cellular myelocytomatosis oncogene; COX-2—cyclooxygenase-2; C-PARP—poly(ADP-ribose) polymerase-1 cleavage; CypB—cyclophilin; ECM—extracellular matrix; eHDFs—embryonic human dermal fibroblasts; EI—erythema index; ERK—extracellular signal-regulated kinase; FAK—focal adhesion kinase; FDPs—fine dust particles; GCLC—glutamate cysteine ligase catalysis subunit; GCLM—glutamate cysteine ligase modifier subunit; *GLT25D1*—gene encoding collagen galactosyltransferase 1; H_2_O_2_—hydrogen peroxide; HaCaT—spontaneously transformed human keratinocyte cell culture; HA—hyaluronic acid; HDFs—human dermal fibroblasts; HDFα—adult human dermal fibroblast; HO-1—heme oxygenase 1; hsa-miR—homo sapiens microRNA; HSP47—heat shock protein 47; IGF-1—insulin-like growth factor 1; IGF-1R—insulin-like growth factor 1 receptor; IGF—insulin-like growth factor; IL—interleukin; iNOS—inducible nitric oxide synthase; IRAK1—IL-1 receptor-associated kinase; IRS—insulin receptor substrate; IκB—inhibitor of NF-κB; JNK—Jun N-terminal kinase; Keap-1—Kelch-like ECH-associated protein 1; MAPK—mitogen-activated protein kinase; MMP—matrix metalloproteinase; MP—methylparaben; MT1-MMP—membrane-type 1 matrix metalloproteinase; mTOR—mammalian target of rapamycin; NF-κB—nuclear factor-kappa B; nHDFs—newborn human skin fibroblasts; NQO-1—NAD(P)H quinone dehydrogenase 1; Nrf-2—nuclear factor erythroid 2-related factor 2; OI—osteogenesis imperfecta; OVX—ovariectomized; p38—p38 mitogen-activated protein kinase; pAkt—phosphorylated protein kinase B; pAMPK—phosphorylated adenosine monophosphate-activated protein kinase; pc-Fos—phosphorylated c-Fos protein; pc-Jun—phosphorylated c-Jun protein; PDI—protein disulfide isomerase; PDK1—phosphoinositide-dependent kinase; pERK—phosphorylated extracellular signal-regulated kinase; PI3K—phosphoinositide 3-kinase; pJNK—phosphorylated Jun N-terminal kinase; PMs—particulate matters; pmTOR—phosphorylated mammalian target of rapamycin; pNF-κB—phosphorylated nuclear factor-kappa B; pNrf-2—phosphorylated nuclear factor erythroid 2-related factor 2; pp38—phosphorylated p38 protein; PP—propylparaben; pSmad—phosphorylated suppressor of mothers against decapentaplegic; rER—rough endoplasmic reticulum; ROS—reactive oxygen species; SASP—senescence-associated secretory phenotype; SC—stratum corneum; SD—Sprague Dawley; SIRT 3—Sirtuin 3; Smad—suppressor of mothers against decapentaplegic; SOD—superoxide dismutase; Sp1—specificity protein 1; STAT—signal transduction and activation of transcription; TEWL—transepidermal water loss; TGF-β—transforming growth factor β; TIMP—tissue inhibitors of metalloproteinase; TNF—tumor necrosis factor; TβRI—type I receptor for transforming growth factor β; TβRII—type II receptor for transforming growth factor β; UV—ultraviolet; γ-GCLC—γ-glutamate-cysteine ligase; γ-H2AX—gamma histone family member X.
